# Strategies to Make Ramadan Fasting Safer in Type 2 Diabetics: A Systematic Review and Network Meta-analysis of Randomized Controlled Trials and Observational Studies

**DOI:** 10.1097/MD.0000000000002457

**Published:** 2016-01-15

**Authors:** Shaun Wen Huey Lee, Jun Yang Lee, Christina San San Tan, Chee Piau Wong

**Affiliations:** From the School of Pharmacy, Monash University Malaysia, Bandar Sunway (SWHL, JYL), School of Allied Health Sciences, SEGi University, Kota Damansara (CSST); and Jeffery Cheah School of Medicine and Health Sciences, Monash University Malaysia, Bandar Sunway, Malaysia (CPW).

## Abstract

Ramadan is the holy month for Muslims whereby they fast from predawn to after sunset and is observed by all healthy Muslim adults as well as a large population of type 2 diabetic Muslims.

To determine the comparative effectiveness of various strategies that have been used for type 2 diabetic Muslim who fast during Ramadan.

A systematic review and network meta-analysis of randomized controlled studies (RCT) as well as observational studies for patients with type 2 diabetes who fasted during Ramadan was conducted. Eight databases were searched from January 1980 through October 2015 for relevant studies. Two reviewers independently screened and assessed study for eligibility, assessed the risk of bias, and extracted relevant data. A network meta-analysis for each outcome was fitted separately, combining direct and indirect evidence for each comparison.

Twenty-nine studies, 16 RCTs and 13 observational studies each met the inclusion criteria. The most common strategy used was drug changes during the Ramadan period, which found that the use of DPP-4 (Dipeptidyl peptidase inhibitor -4) inhibitors were associated with a reduction in incidence of experiencing hypoglycemia during Ramadan in both RCTs (pooled relative risk: 0.56; 95% confidence interval: 0.44–0.72) as well as in observational studies (pooled relative risk: 0.27; 0.09–0.75). Ramadan-focused education was shown to be beneficial in reducing hypoglycemia in observational studies but not RCTs (0.25 versus 1.00). Network meta-analyses suggest that incretin mimetics can reduce the risk of hypoglycemia by nearly 1.5 times.

The newer antidiabetic agents appear to lower the risk of hypoglycemia and improved glycemic control when compared with sulfonylureas. Ramadan-focused education shows to be a promising strategy but more rigorous examination from RCTs are required.

## Abstract 2

Supplemental Digital Content is available in the text

## INTRODUCTION

Diabetes is a fast becoming a global health problem with devastating human, social, and economic impact. Recent estimates suggest that approximately 382 million people worldwide are living with diabetes, representing a prevalence of 8.3% and estimates in several large Muslim majority countries suggest that the prevalence are even higher. In a recent 2010 demographic study, approximately 1.6 billion or 23% of the worldwide population are followers of Islam, and is growing by ∼3% per year.^1^ Fasting during Ramadan, a holy month of Islam, is an obligatory duty for all healthy adult Muslims, whereby they abstain from eating, drinking, use of oral medications, and smoking.^2^ It can be considered as a period of “intermittent fasting” or daily cycles of “alternating” fasting and feeding periods lasting between 11 and 19 hours a day depending on geographical location during 28 to 30 days.^3^ The act of fasting during Ramadan is only obligatory to adults who are healthy with exceptions for certain groups, such as Muslims with serious illnesses, the elderly, travelers, and expecting and nursing mothers.^2,3^

Despite that, many Muslim diabetics choose to fast during this period and this poses some obvious concern and challenge for the Muslim individual as well as their health-care practitioners. Such practices can be seen in the epidemiology of diabetes and Ramadan study, which was conducted in 13 Muslim countries with almost 12,914 diabetics showed that 43% of patients with type 1 and 79% of patients with type 2 diabetes mellitus (T2DM) fasted during Ramadan,^2^ leading to an estimate that worldwide more than 80 million people with diabetes fast during Ramadan. The recent epidemiology of diabetes and Ramadan study also showed that the risk of hypoglycemia is increased by 7.5 times in type 2 diabetics during Ramadan.^2^ Despite the large number of Muslim diabetics fasting, there is no clear scientific consensus on the strategies or advice that should be given for patients who are fasting. This systematic overview aims to determine the strategies that have been successfully used for type 2 diabetics who wish to fast during Ramadan.

## METHODS

### Data Sources and Search Strategy

We conducted a systematic search for studies (randomized or observational) in type 2 diabetes patients who wish to fast during Ramadan. The following electronic databases were searched from inception to October 30, 2015: PubMed, Ovid, MEDLINE, Embase, Cochrane Central Register of Controlled Trials, PsychINFO, CINAHL Plus, Electronics Thesis Online Services as well as ClinicalTrials.gov using a combination of both MesH descriptors as well as free text terms to identify for relevant studies. This was supplemented by manual search of references from identified articles to widen the search results. To ensure a comprehensive search publication search was not restricted on languages or type of publications. Ethical approval was not sought, as this study was based upon published data.

### Study Selection and Quality Assessment of Studies

Two reviewers independently screened the titles, abstracts, and full-text reports to confirm for eligibility. A primary study was eligible if examined people with type 2 diabetes who are fasting during the month of Ramadan; had a comparison of the effect of either a drug, supplement, behavioral or lifestyle therapy, or counseling; and reported 1 or more of the following outcomes: hypoglycemia, weight change, or biochemical data. Any duplicates found within databases were crosschecked and the most recent publications were included in this study.

One reviewer extracted all relevant information from identified articles, which was independently verified by a second reviewer using a standard extraction template developed specifically for this study. The strength and quality of identified publication was assessed using the Jadad score and Cochrane risk of bias assessment tool for randomized controlled studies (RCTs), whereas the Newcastle–Ottawa scale was used for observational studies. Any disagreements between the reviewers were documented and resolved through discussion.

For each study, we extracted the inclusion and exclusion criteria, baseline characteristics for the different treatment arms, intervention and comparators, follow-up period and participants at end of study, and the definition of hypoglycemia as reported in original article. For all treatment arms, we abstracted number of hypoglycemic events experienced during Ramadan; number of patients who experienced hypoglycemia; change in serum fructosamine concentrations, glycated haemoglobin (HbA1c) levels, body mass index, fasting blood glucose; and number of adverse events where reported. In the event of missing information, an attempt was made to contact study authors.

### Data Analyses

We assessed the patient characteristics and outcomes for clinical heterogeneity and used the I^2^ statistics to quantify the total variations because of statistical heterogeneity. When we found heterogeneity, we attempted to determine the potential reasons by examining the characteristics of each individual study. Results are summarized as pooled relative risk (RR) and 95% confidence intervals (CI) for dichotomous outcomes and as rate ratios with 95% CI for event rates. For continuous data, we calculated the differences between baseline and end of study for RCTs and pre- and postdata of the treated cohort for observational studies. In the event of unavailability of standard deviations, we calculated the variance using a standard formula, inputting a correlation coefficient taken from the largest study available.^4^ Any data provided as median and range were converted to mean and standard deviation using appropriate formula as described elsewhere. We used the random effects assumption throughout the study.

We subsequently conducted a random effect network meta-analysis when sufficient data on the same outcome were available (minimum 5 studies). The evidence was summarized using a network diagram assuming common heterogeneity among studies.^5^ Consistency between direct and indirect evidence was assessed using design by treatment interaction model and inconsistency using the loop-specific method. Results of the network meta-analysis are presented as their 95% credible intervals. We subsequently used the surface under curve cumulative ranking curve to rank treatments.^6^ Publication bias was assessed visually using a funnel plot based upon Begg and Eggar method. A sensitivity analysis was conducted using alternative effect measures (odds ratio versus RR) as well as consideration on heterogeneity (random versus fixed effects). Pair-wise analyses were performed using Review Manager 5.3 (Cochrane Collaboration, Oxford, UK), whereas network meta-analysis was conducted using the mvmeta command in Stata (StataCorp, College Station, TX). A *P* value of less than 0.05 was considered significant, and I^2^ value of >50% indicated statistical heterogeneity.

### Sources of Funding

The authors declare that no funding was received for this study. This study is registered with International prospective register for systematic review under the registration no CRD42014013665.

## RESULTS

### Study Selection

**FIGURE 1:**
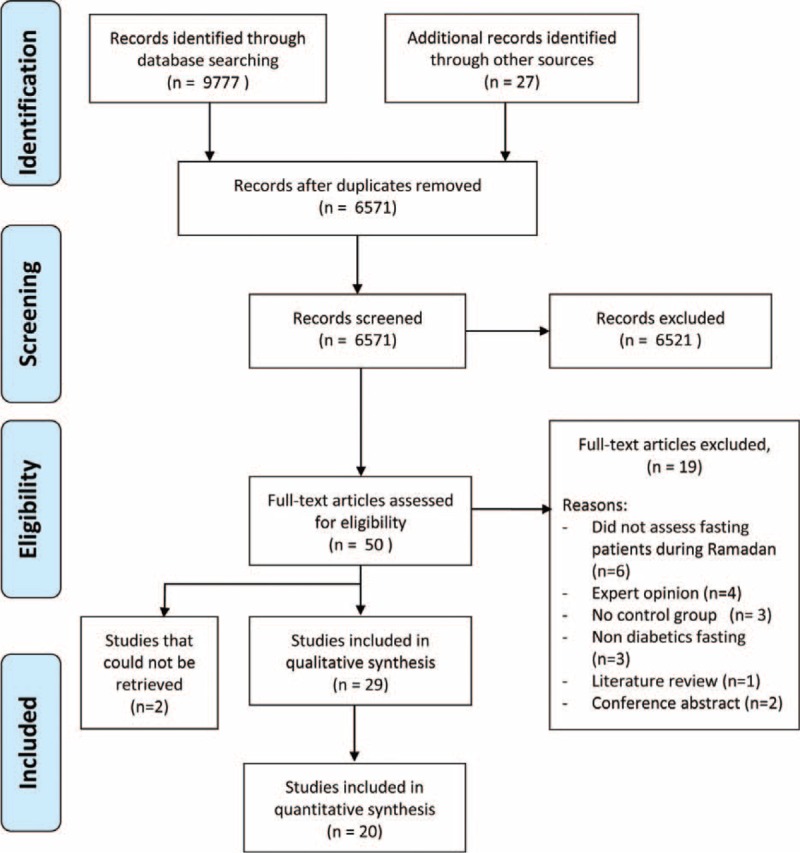
Flow diagram depicts article selection process.

The search yielded a total of 6571 studies and 50 that underwent full-text review (Figure 1). Two full-text articles could not be retrieved.^7,8^ A total of 29 articles met the inclusion criteria and these include 16 RCTs and 13 observational studies (Supplementary Tables 1 and 2). Twenty studies were subsequently included for the meta-analysis. Twenty-one studies (12 RCTs and 9 observational studies) examined the effects of changing drug therapy during Ramadan^9–29^ and 4 studies compared the effects of a Ramadan-focused education^27–33^ (2 RCTs and 2 observational studies). Two studies determined the effects of drug adjustment during Ramadan period,^34,35^ 1 observational study compared the effects using meglitinides during Ramadan,^36^ whereas another study determined the effects of remote telemonitoring.^37^ Hypoglycemia was defined as symptoms (without any threshold) in 6 studies, whereas in another 17 studies, as symptoms coexisting with low blood glucose levels (minimum threshold 2.8 mmol/L, maximum 3.9 mmol/L). Demographic aspects of included studies are summarized in Table 1.

**TABLE 1:**
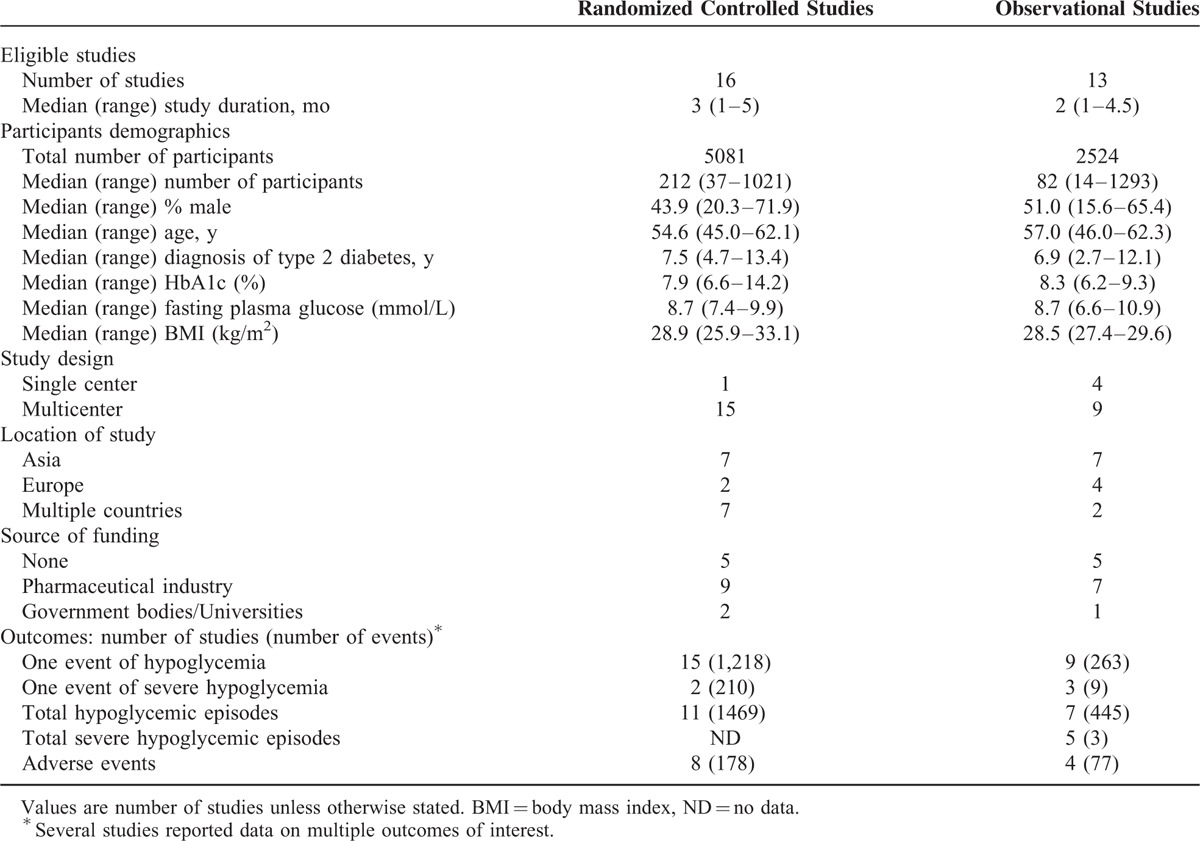
Summary of Baseline Characteristics of Studies Included

The quality of included RCT studies varied, as most studies had an unclear risk of bias in 3 to 5 of the 7 items assessed. Only 9 studies reported adequately the method of random sequence generation, 4 described the detailed method of allocation concealment, whereas only 1 blinded patient caregivers and outcome assessors. For observational studies, apart from lack of information on dropouts, most of the quality rating criteria were met.

### Results for Randomized Controlled Trials

Table 2 summarizes the results of the 16 trials. Meta-analyses shows a lower risk of experiencing at least 1 hypoglycemic events in patients taking an active comparator drugs compared with those receiving Sulfonylurea (SU) (RR: 0.60; 95% CI: 0.48–0.74) during Ramadan. Subgroup analysis by classes of drug showed that only incretin mimetics were associated with a lower rate of causing hypoglycemia during Ramadan (RR: 0.56; 0.44–0.72), but not other active comparators. Further stratifying the group to examine only DPP-4 inhibitors by exclusion of the study by Brady et al^15^ resulted in no difference in the rates of hypoglycemia (RR: 0.56; 0.43–0.74). In studies comparing different insulin formulation, there was no apparent difference between insulin lispro and insulin 30/70 (RR: 0.97; 0.75–1.24). Similarly, no differences were found in hypoglycemia rates when comparing a thiazolidinediones (TZD) compared with placebo or Ramadan-focused education versus usual care. For studies that reported the total hypoglycemic episodes, there was a lower rate of hypoglycemia in patients taking other active comparators (RR: 0.82; 0.70–0.97) compared with SU. Subgroup analysis similarly revealed that only incretin mimetics were significantly associated with a reduced rate of hypoglycemia (RR: 0.83: 0.70–0.98). In the other 2 studies that compared the use of different insulin formulations, reported hypoglycemic episodes were lower with insulin lispro (rate ratio: 0.87; 0.61–1.23).

**TABLE 2:**
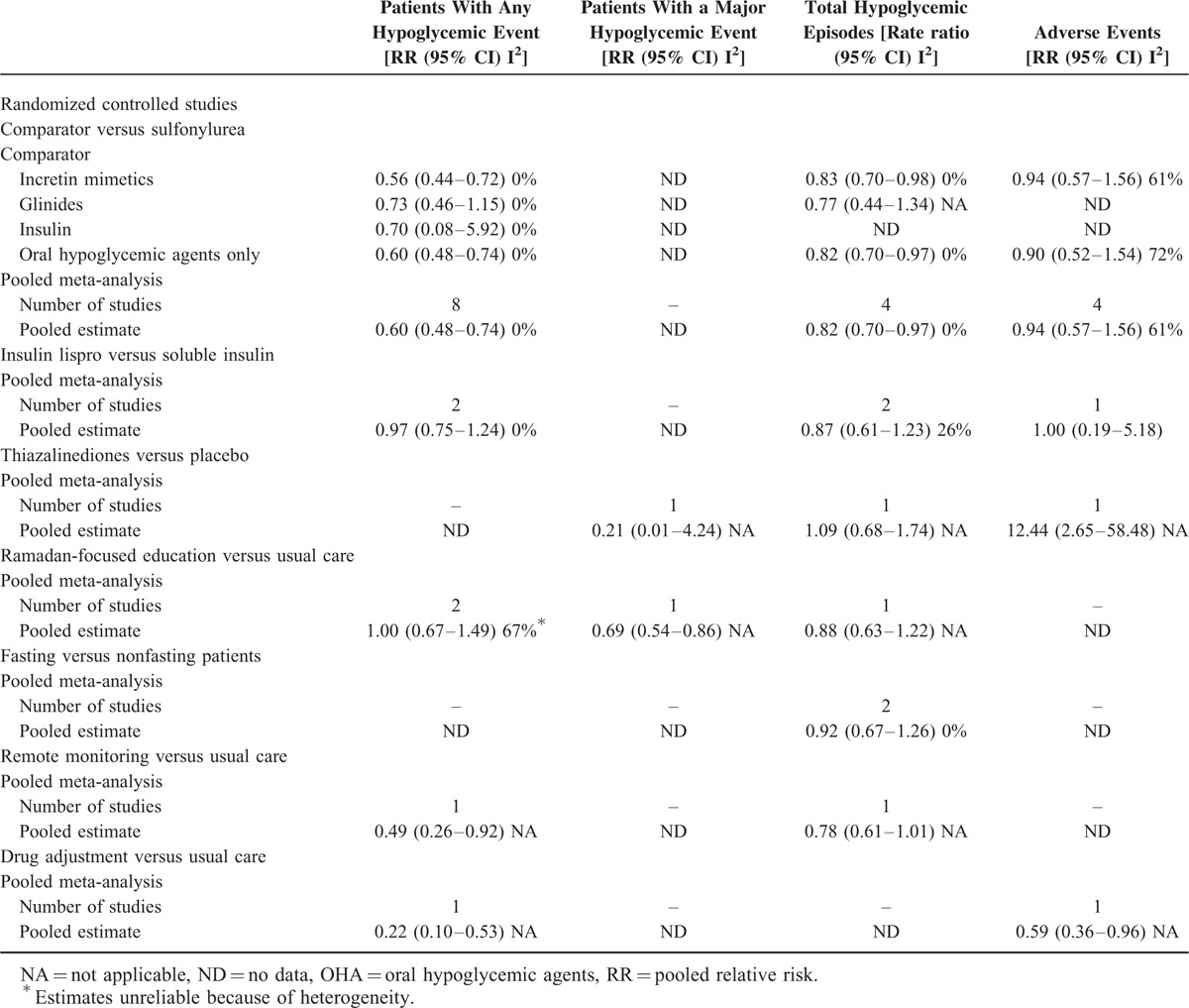
Meta-analyses Summary Estimates of Randomized Controlled Studies on the Impact of Various Strategies Used During Ramadan

Glycemic control during Ramadan was assessed in 10 studies. Pooled analysis of studies that reported serum fructosamine levels showed that glycemic control was better with the use of meglitinides compared with SU [weighted mean difference (MD): −9.97 mg/dL; 95% CI: −11.20 to −8.74]. Similarly, meta-analysis of the 2 studies comparing different insulin formulations showed a trend toward better control with insulin lispro, with a mean difference of 0.21 mmol/L in the fasting plasma glucose levels (−0.31 to −0.11, *P* < 0.001). Reported change in HbA1c showed a small effect in the direction of improved glycemic control with the use of SU (MD: 0.12%; −0.14 to 0.37); however, the results were heterogeneous (I^2^: 99%, *P* < 0.001) and therefore should be interpreted with caution. Subgroup analysis revealed that this was because of different class of agents used, as meglitinides was associated with worse glycemic control. Exclusion of this class of drugs reduced the heterogeneity to 50% and showed a decrease in HbA1c with the use of incretin mimetics (MD: −0.05; −0.38 to 0.27).

Eight studies reported the adverse events that occurred during the course of study. Meta-analysis of the 4 studies, which had a common comparator showed that there was no significant difference between incretin mimetics versus SU (RR: 0.94; 0.57–1.56). In the RCT examining the use of TZD during Ramadan, a higher number patients receiving TZD reported ankle edema. Patients who were treated with the combination of insulin detemir and biphasic insulin reported lower rates of adverse events, whereas the adverse event rates were similar between the 2 insulin preparations.

### Network Meta-analysis of Randomized Controlled Studies

**FIGURE 2:**
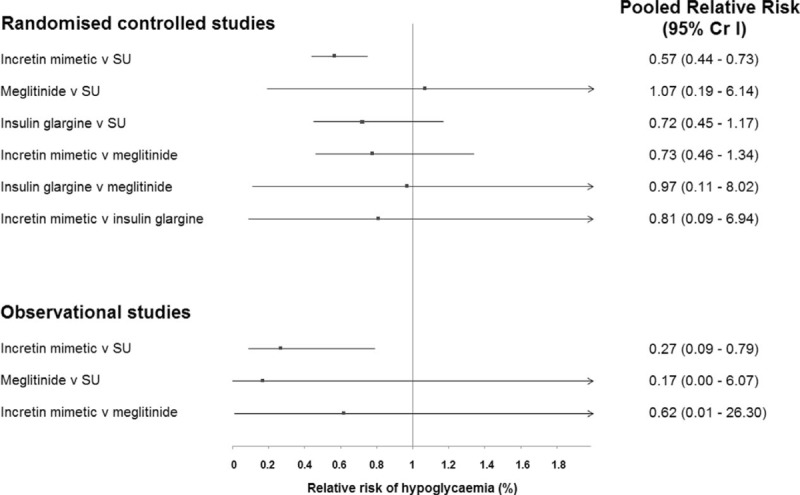
Summary estimates from the network meta-analysis on risk of hypoglycemia in patients fasting during Ramadan using different pharmacological agents. CrI = credible interval.

Network meta-analysis on hypoglycemic events showed that use of incretins was associated with a lower rate of causing hypoglycemia (RR: 0.57; 0.44–0.73). The remaining treatments, however, were not statistically significant in our network meta-analysis (Figure 2). Based upon the surface under cumulative ranking curve, incretin mimetics were the most effective treatment in reducing the rates of hypoglycemia in T2DM patients fasting during Ramadan, followed by insulin glargine, meglitinides, and finally SU (Supplementary Figure 1). Results for direct and indirect meta-analysis were consistent statistically.

Results suggest that there was little evidence of publication bias in the RCT studies (Supplementary Figure 2). Sensitivity analysis using alternative effect measures (RR versus odds ratio) as well as considerations of heterogeneity (random effect versus fixed effect) did not show any important changes in the pooled effects except for studies examining Ramadan focused education; and therefore, results should be interpreted with caution (Supplementary Table 3).

### Study Outcomes for Observational Studies

Overall, results were comparable with those found in the RCTs, suggesting that the use of incretin mimetics, specifically DPP-4 inhibitors were associated with a lower risk of hypoglycemia (Table 3). Meta-analyses yielded a reduction in patients experiencing hypoglycemia (RR: 0.27; 0.09–0.75) or severe hypoglycemia (RR:0.33; 0.09–1.15) as well as total hypoglycemic events (rate ratio: 0.29; 0.08–1.04), which is better than RCTs. Heterogeneity in the overall meta-analysis on the risk of experiencing any hypoglycemic event was noted, mainly because of the study by Halimi et al,^18^ which was the only study that had reported no significant difference in their intervention. Exclusion of this study removed heterogeneity and produced a slightly higher reduction in risk of hypoglycemia (RR: 0.23; 0.16–0.34). In addition, meta-analyses of observational studies on Ramadan-focused education suggest that this could reduce the rates (RR:0.25; 0.09–0.67) as well as total hypoglycemic events (rate ratio: 0.78; 0.23–2.68).

**TABLE 3:**
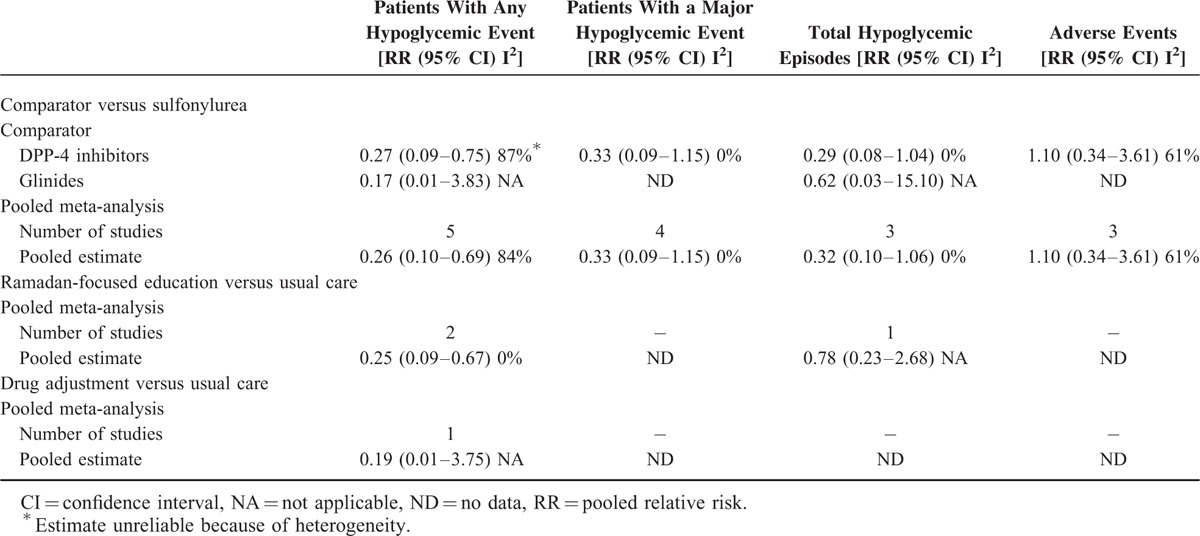
Meta-analyses Summary Estimates of Observational Studies on the Impact of Various Strategies Used During Ramadan

In studies that reported a change in HbA1c after Ramadan, use of other agents besides SU resulted a decrease of 0.09% (−0.28 to 0.11) in HbA1c compared with sulfonylurea. Only 6 studies reported the change in weight 1 month post Ramadan. Studies found that the use of DPP-4 inhibitors resulted in a small but nonsignificant reduction in weight compared with those treated with SUs (MD: −0.51; −4.15 to 3.13). Three studies reported adverse events associated with their intervention, but there was substantial heterogeneity in the strengths of estimated RR.

### Network Meta-analysis of Observational Studies

Consistent with our results from RCT studies, network meta-analysis on hypoglycemic events showed that only the use of incretins was associated with a lower rate of causing hypoglycemia (RR: 0.27; 0.09–0.79; Figure 2). Ranking on efficacy of treatment based upon probability suggest that incretins was the most effective treatment in reducing the rates of hypoglycemia, followed by meglitinides, and finally SU (Supplementary Figure 3). Owing to the small number as well as variation in study characteristics, publication bias was not assessed formally. Funnel plot, however, suggests asymmetry, suggesting the presence of bias (Supplementary Figure 4). Sensitivity analysis suggests that the magnitude of reduction in hypoglycemia was smaller but was not statistically significant in most cases (Supplementary Table 4).

## DISCUSSION

Fasting during Ramadan for patients with type 2 diabetes carries a risk of an assortment of complication. The most fearful among all is the risk of hypoglycemia, which is a major concern for most patients and practitioners as there is a change in the timing of fluid and food intake alongside reduction in meal frequency. As such, most recommendations currently advocate that patients should have ample discussion with their health-care practitioners on the risk and benefits of fasting.^38,39^ In patients who wish to fulfill their religious obligations and fast, they should be stratified according to their risk of hypoglycemia and/or presence of complications as recommended by International Group for Diabetes and Ramadan.^40^ Indeed, as shown by results of this study, it is possible for patients with T2DM to fast, as the rates of hypoglycemia are relatively similar between those who fast and do not fast during Ramadan.

In patients who fast during Ramadan, drug class changes were the most common strategy used to prevent hypoglycemia. Results from both pair-wise as well as network meta-analysis of RCTs and observational studies suggest that the use of incretin mimetics, specifically DPP-4 inhibitors were associated with the lowest incidence and rate of hypoglycemia, when compared with sulfonylureas. Our findings are consistent with results of other studies, which have shown incretin mimetics to be associated with lower rates of hypoglycemia.^41,42^ Owing to the width of the credible interval, which was considerable, we, however, urge caution in the interpretation of ranking and probability of these treatment being the “best.” In particular, we note the uncertainty around the use of meglitinides, which there was insufficient evidence to draw a firm conclusion over its efficacy compared with SU.

Adding to the quagmire is the use of insulin in type 2 diabetics who fast during Ramadan, because of the increase risk of hypoglycemia compared with oral agents.^43,44^ Similar to the strategy used in oral hypoglycemic agents, most studies in this review switched their patients to an intermediate acting insulin preparation, which has a lower propensity to cause hypoglycemia. Results from 3 RCTs found that it was safe to fast during Ramadan while on insulin, and the use of an intermediate-acting insulin lispro compared insulin 30/70 reduced the number of hypoglycemic events by nearly a quarter. Unfortunately, given the limited number of studies, its impact and safety needs to be further tackled in future research.

Besides drug changes, studies have shown that educational session can be an effective strategy in managing patients with T2DM, either alone or in combination with other pharmacological therapies.^37,45^ Ramadan-focused education have been found to empower patients and increase their self-care awareness, leading to a lower risk of hypoglycemia while helping with glycemic control.^33^ Results of this study together with previous meta-analysis on behavioral/educational interventions^45^ suggest that educational session should be provided to all T2DM patients who wish to fast either individually or in groups, and focus on issues, such as risk of fasting, meal planning, timing, and dosing of medication among others. Such educational session could even be incorporated as part of the public health messages provided through Imans in mosque during this period. A new study examining the potential of combining Ramadan-focused education (NCT02189135), self-monitoring of glucose and diet in fasting Ramadan patients if proven successful may have wide public health implications.

Our systematic also review reveals a paucity of studies that are available on strategies for type 2 diabetic Muslims to fast safely during Ramadan. This could be attributed to the difficulties in recruitment, as there is only a limited time frame available to conduct the study during Ramadan. During this study, we also have found that there is a divergence in outcome measurements reported from various studies. This is largely because of the lack of a standard protocol or recommendations in reporting outcome measurement in diabetic patients who fast during Ramadan. As such, we would recommend that future studies on Ramadan should include a nonfasting group as control, conducted during a period of 3 months (1-month pre-Ramadan and 1-month post) in various geographical locations, and report glycemic control using HbA1c, serum fructosamine as well as fasting plasma glucose alongside with incidences and rates of hypoglycemia in their cohort. This would allow clinicians a common ground to compare the efficacy of the treatment while balancing the need for safety.

### Strengths and Weakness of Current Study

Our study has several strengths. To our knowledge, this is the first and most comprehensive systematic review and network meta-analyses, which have addressed this important issue on strategies used to ensure diabetic Muslims, can fast safely during Ramadan. This is complemented by a comprehensive search strategy, without any language restriction. Although we attempted to retrieve all relevant articles, it remains possible that relevant studies were missed. Our study also summarizes the experiences of different populations from various countries, which may represent the diverse and heterogeneous Muslim population of varying geographical distribution, lifestyles, and habits. We also had 2 authors who independently extracted all data and obtained or confirmed data with corresponding authors from included studies whenever possible. During this work, we found that another meta-analyses exclusively focusing on noninsulin glucose-lowering agents were published.^46^ Despite the difference in inclusion and exclusion criteria, the results were remarkably consistent. In addition, given the lack randomized studies available, our approach of a systemic joint presentation of both results from both types of studies is required to provide the most comprehensive summary of evidences available to date.

This study has some limitations. The method of randomization and allocations were seldom described in the studies reported. Similarly, a lack of allocation concealment may significantly influence the results. We had also assessed people with type 2 diabetes only, which may limit the ability to generalize our findings to a wider audience including people with type 1 diabetes. There were also only a few studies, which had contributed to the network meta-analysis that limited our ability to draw firm conclusions. There was also heterogeneity in the design of observational studies as well as the definition and reporting of results from both randomized controlled studies and observational studies. Also, differences in reported hypoglycemia between studies maybe because of the duration of fast can vary between 10 and 18 hours depending on the time of the year and latitude, which could not be independently assessed in our network meta-analysis. In addition, many trials were not powered to assess our predefined outcomes, and as such increases the risk of reporting bias.

## CONCLUSIONS

With more than 50 million Muslims worldwide with T2DM, health-care professionals worldwide would need to be aware of the risk and benefits of fasting during Ramadan and provide Ramadan specific diabetes care. Hypoglycemia represents one of the greatest health concerns for both patients and caregivers, and results of this study shows that it is possible for T2DM patients to fast during this period. Findings of this study suggest that the use of educational initiatives can be beneficial for those wishing to fast during Ramadan. In patients who have a high risk of experiencing hypoglycemia, these patients should be advised and educated on the risk of fasting and if they insists of fasting, agents with a low propensity to induce hypoglycemia such as the incretin mimetics should be considered. In addition, in T2DM patients treated with insulin, the choice of analogue maybe more appropriate given the lower risk of hypoglycemic events and postprandial glucose control.

In terms of research, future studies should also focus on studying the effects of different health-care support strategies or even self-management intervention to provide new information for clinicians on the best strategies to manage diabetes during Ramadan. It should also contain a standardized outcome measure and include an economic evaluation of antidiabetic drugs used or replaced during the month of Ramadan. Finally, to disclose the full potential benefits of such intervention, a continued follow-up of the randomized controlled studies are necessary.

## Supplementary Material

Supplemental Digital Content
