# Supplementary material for: Strategies to Make Ramadan Fasting Safer in Type 2 Diabetics: A Systematic Review and Network Meta-analysis of Randomized Controlled Trials and Observational Studies
Source: Medicine (Baltimore). 2016 Jan 15;95(2):e2457. doi: 10.1097/MD.0000000000002457 (PMC4718266; doi:10.1097/MD.0000000000002457)
Supplement: Supplemental Digital Content [file medi-95-e2457-s001.pdf]

Supplemental Figure 1: Surface under the cumulative ranking curve for risk of hypoglycaemia in randomized controlled studies. Larger SUCRA value suggests better results for the respective intervention

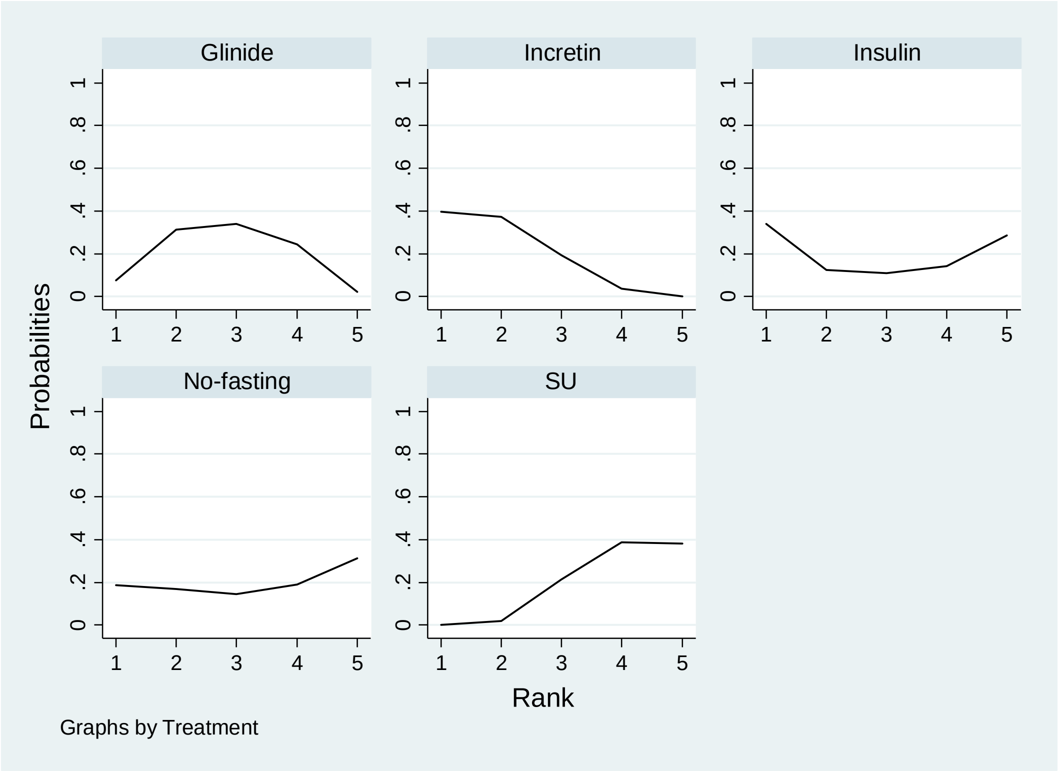

Supplemental Figure 2: Funnel plot depicting randomized controlled studies which examine sulfonylureas compared to other interventions

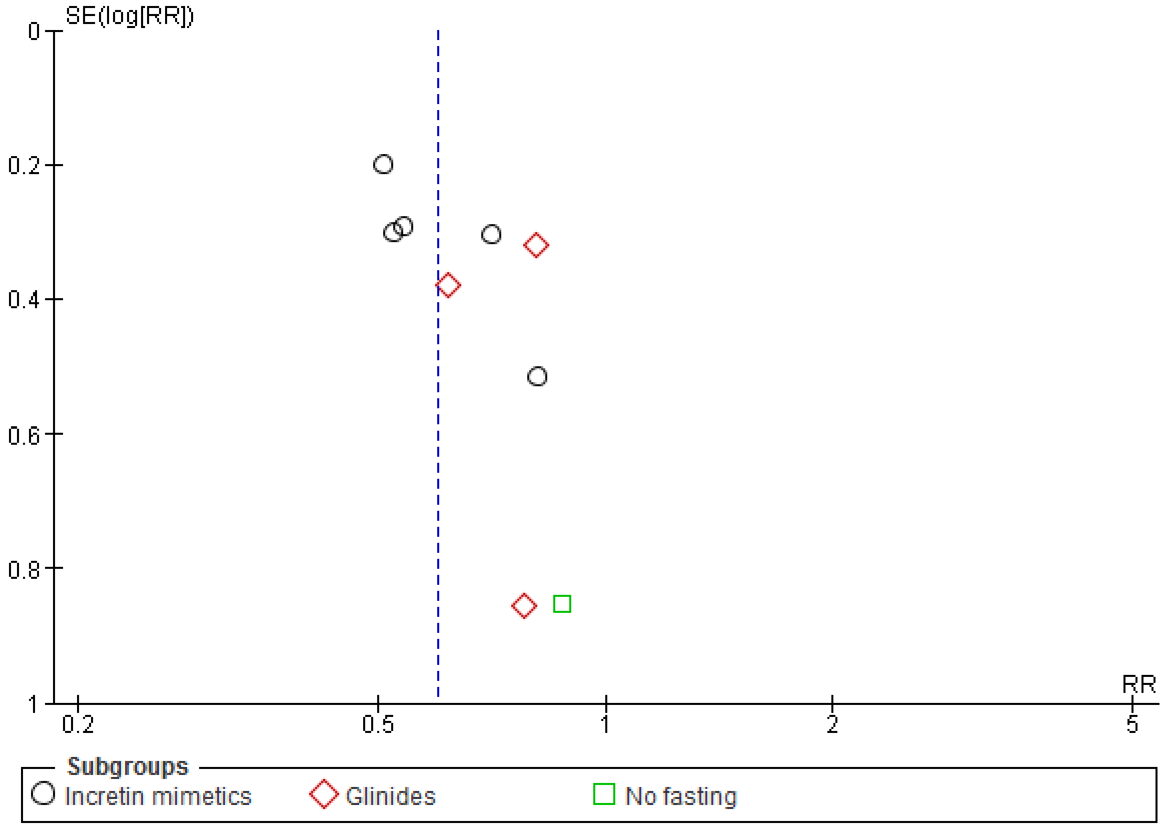

Supplemental Figure 3: Surface under the cumulative ranking curve for risk of hypoglycaemia in observational studies. Larger SUCRA value suggests better results for the respective intervention

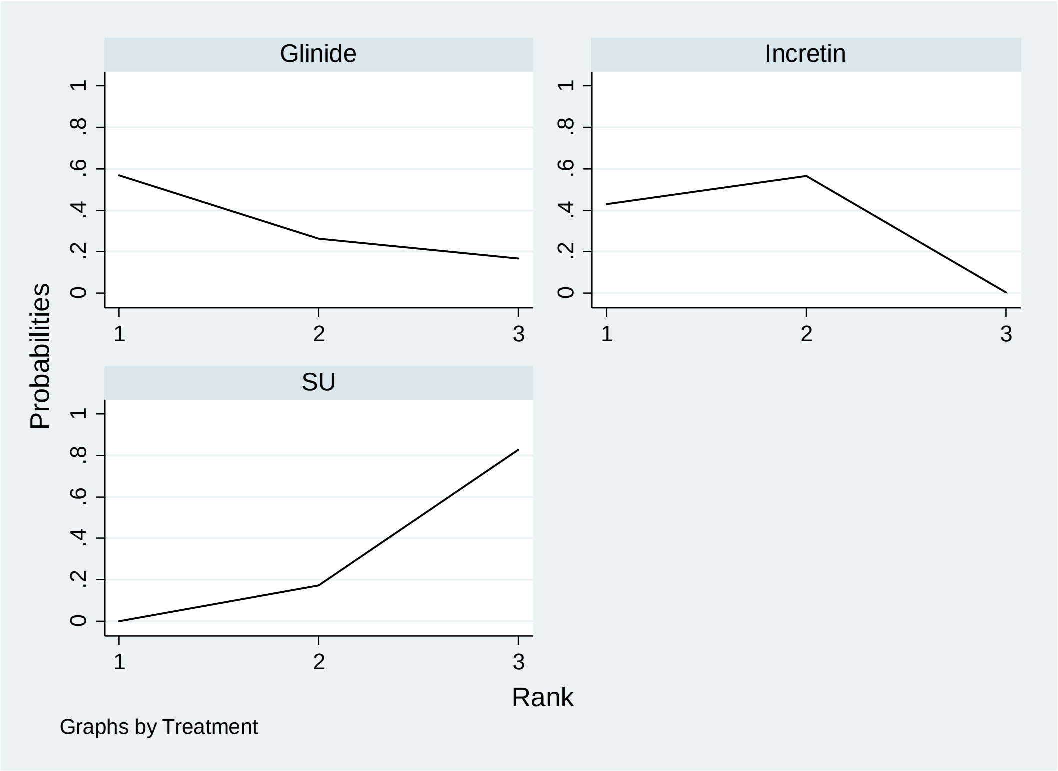

Supplemental Figure 4: Funnel plot depicting observational studies which examine sulfonylureas compared to other interventions

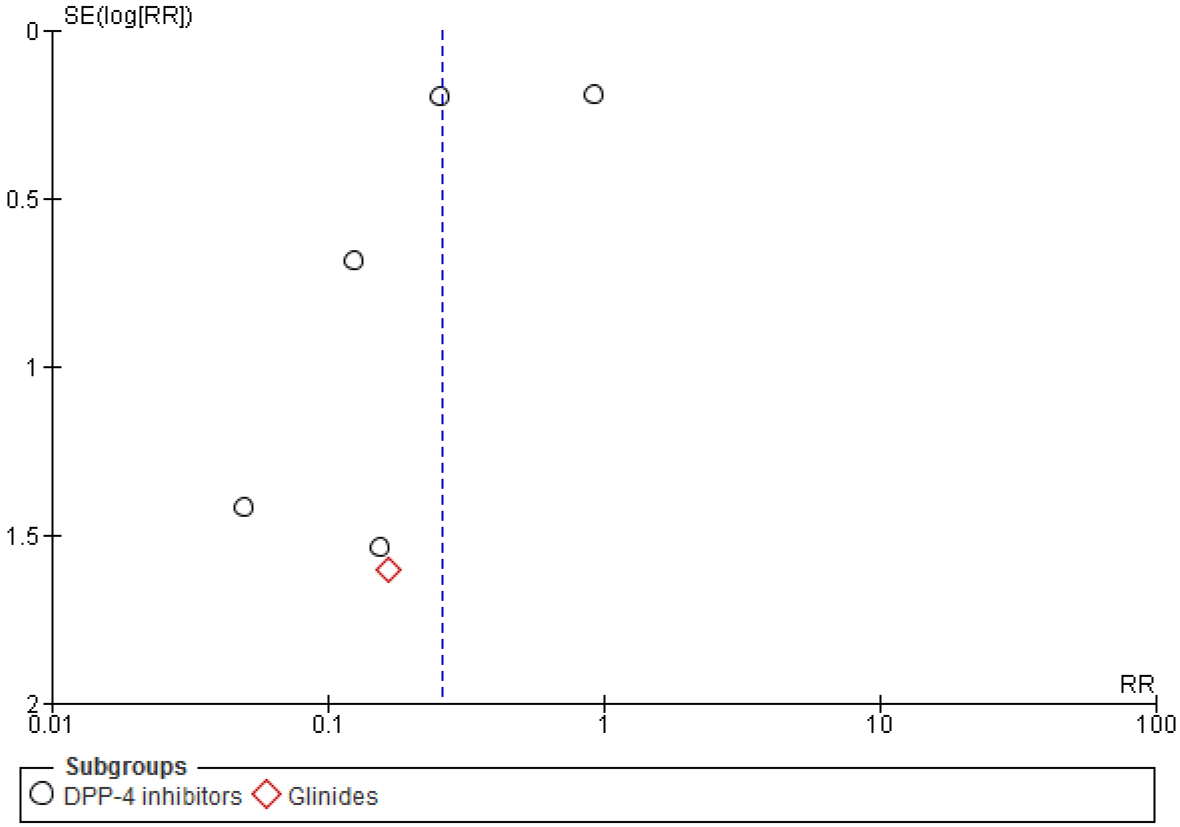

Supplemental Table 1: Baseline characteristics of randomized controlled studies included in the review

Author, year	Intervention	Total patient recruited (n)	Countrie(s)	Setting	Age, mean ± SD (years)	No of males, n (%)	Duration of diabetes, mean ± SD (years)	BMI, mean ± SD (kg/m^2)	HbA1c, mean ± SD (%)	Fasting blood glucose, mean ± SD (mmol/L)
Akram, 1999	Insulin lispro	68	Egypt, Saudi Arabia, Kuwait, United Arab Emirates, Pakistan	Multi-center	50.3 ± 1.1	38 (55.9)	11.6 ± 0.7	28.3 ± 0.6	NR	NR
	Soluble insulin 30/70									NR
Al-Sifri, 2011	SU	514	Egypt, Israel, Jordan, Lebanon, Saudi Arabia, United Arab Emirates	Multi-center	55.0 ± 10.0	255 (49.6)	6.0[+]	30.5 ± 5.6	7.6 ± 1.2	8.5 ± 2.8
	DPP-4 Inhibitor	507			55.0 ± 11.0	269 (53.1)	5.0[+]	30.5 ± 5.7	7.5 ± 1.3	8.3 ± 2.9
Anwar, 2006	Glinide	17	Malaysia	Single-center	5 (38-65)[+]	8 (47.1)	7 (2-20)[+]	NR	7.5 (5.0–13.8)[+]	8.2 (5.9-13.6)[+]
	SU	21			49 (30-74)[+]	8 (38.1)	4 (2-18)[+]	NR	7.6 (6.3-10.2)[+]	7.5 (5.6-13.3)[+]
Aravind, 2012	SU	427	Malaysia, India	Multi-center	50.7 ± 10.0	194 (45.4)	3.0[+]	27.5 ± 4.7	7.9 ± 1.2	NR
	DPP-4 Inhibitor	421			51.4 ± 9.9	208 (49.4)	3.0[+]	27.4 ± 6.0	8.0 ± 1.1	
Belkhadir, 1993	No fasting	194	Morocco	Multi-center	57.2 ± 9.0	56 (28.9)	8.5 ± 6.2	25.9 ± 3.9	14.2 ±3.3	NR
	Full dose SU	199			54.9 ± 9.3	75 (37.7)	7.0 ± 5.5	26.7 ± 3.8	13.7 ±3.4	
	Reduced dose SU	198			54.8 ± 9.7	67 (33.8)	7.1 ± 5.4	26.7 ± 4.2	13.2 ± 3.6	

Brady, 2014	SU	52	United Kingdom	Multi-center	52.2 ± 10.7	26 (50.0)	NR	30.1 ± 4.3	7.8 ± 1.0	NR
	GLP analogue	47			51.5 ± 11.1	24 (51.1)	NR	33.0 ± 7.3	7.6 ± 1.1	
Cesur, 2006	Glinide	18	Turkey	Multi-center	56.3 ± 11.3	10 (55.6)	5.9 ± 4.8	28.9 ± 3.5	6.6 ± 0.8	7.4 ± 1.6
	SU	21			57.4 ± 8.3	15 (71.4)	6.4 ± 5.4	28.5 ± 3.5	6.9 ± 1.3	7.8 ± 1.8
	Glargine	10			54.8 ± 6.0	4 (40.0)	7.1 ± 4.9	28.9 ± 2.0	7.8 ± 0.9	8.8 ± 3.5
Hassanein, 2014	SU	278	Denmark, Egypt, Germany, Indonesia, Jordan, Kuwait, Lebanon, Malaysia, Russia, Saudi Arabia, Singapore, Spain, Tunisia, Turkey, United Kingdom, United Arab Emirates	Multi-center	54.3 ± 9.1	128 (46.0)	4.7 ± 3.8	31.1 ± 5.2	6.9 ± 0.8	7.8 ± 1.9
	DPP-4 Inhibitor	279			54.6 ± 9.3	132 (47.3)	4.8 ± 4.1	30.7 ± 5.0	7.0 ± 0.8	7.9 ± 1.9
Lee, 2015	Usual care	19	Malaysia	Multi-center	47.0 ± 11.0	5 (26.3)	2 (0.7-12.0)*	29.6 ± 6.3	7.4 (5.8-11.0)*	6.8 (4.8-10.6)*

	Remote telemonitoring	18			54.7 ± 8.7	10 (55.6)	10 (0.5-24.0)*	29.0 ± 5.2	8.1 (6.0-12.7)*	6.9 (4.4-15.8)*
Mafauzy, 2002	SU	119	Malaysia, United Kingdom France, Saudi Arabia, Morocco	Multi-center	54.5 ± 6.9	82 (68.9)	7.3 ± 5.0	26.8 ± 3.2	7.7 ± 1.6	8.9 ± 2.4
	Glinide	116			52.7 ± 7.4	87 (75.0)	7.2 ± 4.5	26.5 ± 2.5	8.0 ± 1.7	9.5 ± 2.6
Malha, 2014	SU	39	Lebonan	Multi-center	54.6 ± 9.2	NR	8.4 ± 6.4	28.9 ± 4.5	8.5 ± 1.7	9.5 ± 3.6
	DPP-4 Inhibitor	30			57.0 ± 9.6	NR	9.8 ± 9.2	29.5 ± 4.7	7.9 ± 1.5	8.3 ± 3.0
Mattoo, 2003	Insulin Lispro Mix 25	151	India, Pakistan, Malaysia, Singapore, Egypt, Morocco, South Africa	Multi-center	53.0 ± 9.0	69 (45.7)	12.5 ± 6.5	26.7 ± 3.8	NR	NR
	Soluble Insulin 30/70									
McEwen, 2015	Ramadan focused education	515	Egypt, Iran, Jordan, Saudi Arabia	Multi-center	47 ± 10	209 (40.7)	9 ± 4	NR	NR	NR
	Usual care	259			51 ± 10	106 (41.1)	10 ± 6	NR	NR	NR
Prataksitorn, 2014	Ramadan focused education	108	Thailand	Multi-center	61.1 ± 10.2	19 (17.6)	7.0 ± 4.7	26.4 ± 5.2	9.7 ± 2.7	9.9 ± 3.9
	Usual care	104			62.1 ± 9.2	24 (23.1)	7.5 ± 4.8	26.6 ± 4.7	9.1 ± 2.6	9.2 ± 3.5

Shehadeh, 2015	Drug adjustment	127	Israel	Multi-center	60.1 ± 8.9	50 (39.4)	13.4 ± 6.1	33.0 ± 7.1	8.4 ± 1.0	NR
	Usual care	118			59.4 ± 10.1	44 (37.9)	12.0 ± 5.3	33.1 ± 7.2	8.5 ± 1.1	NR
Vasan, 2006	Placebo	44	India	Multi-center	45.0 ± 9.0	8 (18.2)	8.5 ± 2.2	29.4 ± 5.4	NR	9.0 ± 3.1
	Pioglitazone	43			45.0 ± 9.0	26 (60.5)	8.0 ± 2.0	29.8 ± 5.0	NR	8.7 ± 2.9

Footnotes

HbA1c, hemoglobin A1c; BMI, Body Mass Index; NR: Not reported; [+] Median (range)

Supplemental Table 2: Baseline characteristics of observational studies included in the review

Author, year	Intervention	Total patient recruited (n)	Countrie(s)	Setting	Age, mean ± SD/ range (years)	No of males, n (%)	Duration of diabetes, mean ± SD/ range (years)	BMI, mean ± SD (kg/m^2)	HbA1c, mean ± SD (%)	Fasting blood glucose, mean ± SD (mmol/L)
Al-Arouj, 2013	DPP-4 inhibitor	669	Bangladesh, Egypt, India, Indonesia, Kuwait, Lebanon, Oman, Pakistan, Saudi Arabia, United Arab Emirates	Multi-center	48.0 ± 10.9	386 (57.7)	3.4 ± 3.2	29.4 ± 5.2	7.3 ± 0.8	NR
	SU	624			51.3 ± 10.7	373 (59.8)	4.5 ± 4.3	28.6 ± 5.1	7.4 ± 0.9	NR
Bakiner, 2009	Fasting	7	Turkey	Single-center	50.1 ± 9.7	4 (57.1)	NR	NR	NR	7.8 ± 1.0
	Non-fasting	7			60.6 ± 5.4	4 (57.1)				6.8 ± 1.4
Devendra, 2009	DPP-4 inhibitor	26	United Kingdom	Multi-center	53.2 ± 9.7	16 (61.5)	7.1 ± 3.1	NR	9.0 ± 0.4	NR
	SU	26			62.3 ± 9.8	18 (69.2)	6.9 ± 1.4		9.0 ± 1.3	
Halimi, 2013	DPP-4 inhibitor	115	France	Multi-center	58.4 ± 11.5	73 (63.5)	6.9 ± 4.4	28.1 ± 4.0	7.2 ± 0.6	NR
	SU or glinide	83			59.8 ± 10.3	46 (55.4)	8.1 ± 6.0	28.1 ± 4.8	7.2 ± 0.6	
Hassanein, 2011	DPP-4 inhibitor	23	United Kingdom	Multi-center	58.3 ± 13.1	12 (52.2)	7.1 ± 6.1	29.6 ± 5.0	7.7 ± 0.9	NR
	SU	36			57.3 ± 11.0	21 (58.3)	5.8 ± 4.7	28.5 ± 3.9	7.2 ± 0.6	

Hui, 2010	Humalog Mix 50	26	United Kingdom	Multi-center	62.3 ± 9.8	8 (30.8)	9.8 ± 2.8	NR	9.1 ± 0.4	NR
	Humalog Mix 30	26			61.9 ± 9.2	10 (38.5)	9.5 ± 3.3		9.0 0.4	
Mustafa, 2012	Ramadan focused education	71	United Arab Emirates	Single-center	46.0 ± 17.0	25 (35.2)	NR	NR	8.3 ± 1.9	8.8 ± 2.9
	Usual care									
Norouzy, 2012	Diet	61	Iran	Multi-center	51.0 ± 10.0	45 (51.1)	NR	27.6 ± 3.9	8.2 ± 1.6	8.7 ± 3.5
	OHA	27								
Salti 2009	Insulin naïve	100	Bangladesh, China, Egypt, India, Indonesia, Jordan, Kuwait, Lebanon, Malaysia, Morrocco, Oman, Saudi Arabia, Tunisia, United Arab Emirates	Multi-center	53.5 ± 8.7	50 (50.0)	9.4 ± 6.2	29.4 ± 3.8	9.3 ± 1.4	10.9 ± 3.8
	Insulin treated	249			54.9 ± 9.3	128 (51.4)	12.1 ± 7.4	28.6 ± 4.1	9.1 ± 1.9	9.8 ± 3.9
Sari, 2004	Diet	12	Turkey	Single-center	59 ± 6	NR	2.7 ± 2.1	NR	6.2 ± 0.7	6.6 ± 1.4
	SU	43			57 ± 5		2.9 ± 1.9		6.6 ± 0.9	6.6 ± 1.1
	Glinide	27			58 ± 8		3.3 ± 2.7		7.5 ± 1.0	8.3 ± 2.3

Shete, 2013	DPP-4 inhibitor	55	India	Multi-center	51.0 ± 8.8	NR	NR	NR	8.8 ± 1.3	NR
	SU	42			50.9 ± 9.1				8.6 ± 1.6	
Susiparat, 2014	Ramadan focused education and drug adjustment	62	Thailand	Multi-center	56.3 ± 9.5	9 (30.7)	NR	28.0 ± 6.1	8.7 ± 2.0	8.6 ± 3.5
	Usual care	28			54.8 ± 9.9	5 (17.9)		27.4 ± 2.6	8.9 ± 2.1	9.4 ± 3.3
Wangthong, 2008	Drug adjustment	41	Thailand	Single center	58.6 ± 11.3	10 (24.4)	NR	NR	NR	9.7 ± 2.4
	Usual care	38			57.5 ± 12.2	12 (31.6)				8.7 ± 2.9

Footnotes
HbA1c, haemoglobin A1c; BMI, Body Mass Index; NR: Not reported

Supplemental Table 3: Sensitivity analysis of results using alternative effect measures and statistical model for randomized controlled studies

	Random method	Fixed method
**Hypoglycaemia risk**		
Active comparator vs SU		
Relative risk	0.60 (0.48-0.74)	0.59 (0.48-0.74)
Odds ratio	0.54 (0.42-0.70)	0.54 (0.42-0.70)
Insulin lispro vs Insulin 30/70		
Relative risk	0.97 (0.75-1.24)	0.97 (0.75-1.24)
Odds ratio	0.94 (0.63-1.40)	0.94 (0.63-1.40)
Ramadan focused education vs Usual care		
Relative risk	1.00 (0.67-1.49)	1.13 (1.03-1.24)
Odds ratio	1.11 (0.47-2.62)	1.38 (1.02-1.85)
**Total hypoglycemic episodes (Rate ratio)**		
Active comparator vs SU	0.82 (0.70-0.97)	0.82 (0.70-0.97)
Insulin lispro vs Insulin 30/70	0.87 (0.61-1.23)	0.80 (0.66 -1.18)
**Adverse events**		
Active comparator vs SU		
Relative risk	0.94 (0.57-1.56)	0.91 (0.75-1.10)
Odds ratio	0.89 (0.48-1.64)	0.87 (0.67-1.14)

Supplemental Table 4: Sensitivity analysis of results using alternative effect measures and statistical model for observational studies

	Random method	Fixed method
**Hypoglycaemia risk**		
Active comparator vs SU		
Relative risk	0.26 (0.10-0.69)	0.36 (0.27-0.46)
Odds ratio	0.18 (0.06-0.54)	0.29 (0.21-0.38)
Ramadan focused education vs usual care		
Relative risk	0.25 (0.09-0.67)	0.25 (0.09-0.69)
Odds ratio	0.20 (0.06-0.61)	0.19 (0.06-0.61)
**Severe hypoglycaemia risk**		
Active comparator vs SU		
Relative risk	0.33 (0.09-1.15)	0.29 (0.09-0.97)
Odds ratio	0.32 (0.09-1.14)	0.28 (0.08-0.97)
**Total hypoglycemic episodes (Rate ratio)**		
Active comparator vs SU	0.32 (0.10-1.06)	0.32 (0.10-1.06)
**Adverse events**		
Active comparator vs SU		
Relative risk	1.10 (0.34-3.61)	1.59 (0.99-2.56)
Odds ratio	1.06 (0.29-3.91)	1.63 (1.00-2.66)
